# Analysis of Chip Electronic Components’ Typical Yield in Taping Process Based on Virtual Metrology

**DOI:** 10.3390/s26082292

**Published:** 2026-04-08

**Authors:** Shiqi Zhang, Lizhen Chen, Jiangcheng Fu, Chenghu Yang, Guangli Chen

**Affiliations:** 1 Institute of Electronic Products and Electrical Appliances, Guangdong Academy of Sciences, Guangzhou 510400, China; zhangshq33@alumni.sysu.edu.cn (S.Z.); fjc1921@126.com (J.F.); geri_ych@foxmail.com (C.Y.); giachen2012@163.com (G.C.); 2 Guangdong Electronic Technology Research Institute, Guangzhou 510630, China

**Keywords:** taping, yield prediction, virtual metrology, multidimensional equal-frequency binning, nested cross-validation, Bayesian optimization, imbalanced regression

## Abstract

This study addresses virtual metrology (VM) for the taping process of chip electronic components, in which partial observability, unmeasured disturbances, and severe label imbalance make direct batch-wise yield prediction unstable. Rather than proposing a new standalone learning algorithm, we develop a data-centric VM framework that reformulates the task as the prediction of operating-condition-level typical yield. First, physically relevant features are retained based on process knowledge and analyzed using Pearson correlation, Spearman correlation, and mutual information. We then perform multidimensional equal-frequency binning to partition the observable feature space into locally homogeneous operating condition groups, and define the within-bin median yield as the typical yield, thereby constructing an operating condition dictionary. Based on this dictionary-based representation, low-yield-oriented sample weighting is combined with nested cross-validation and Bayesian optimization for model comparison and hyperparameter tuning. Using desensitized production data from an electronic component taping process, the results under this representation show more stable prediction than direct modeling on unbinned batch samples while also improving tail-oriented fitting relative to unweighted baselines. These findings suggest that, for partially observable manufacturing data, operating condition stratification provides a practical basis for stabilizing VM prediction, while low-yield-oriented sample weighting further improves sensitivity to the low-yield tail, supporting picture yield early warning and process-level decision making.

## 1. Introduction

**Figure 1:**
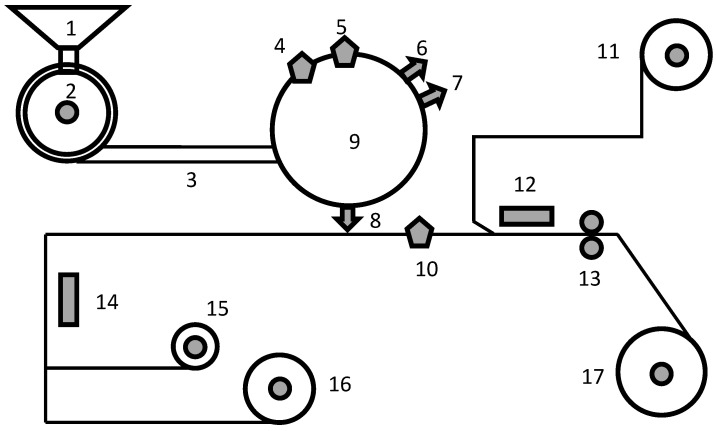
Diagram of taping process. The names of the components are as follows: 1. hopper; 2. vibrating bowl; 3. straight rail; 4. electrical performance testing head; 5. charge-coupled device (CCD); 6. electrical performance NG outlet; 7. picture NG outlet; 8. qualified product outlet; 9. indexing plate; 10. missed-inspection device; 11. upper cover band; 12. upper soldering iron; 13. pressure roller; 14. lower soldering iron; 15. carrier tape; 16. lower cover band; 17. tape-collecting motor.

Chip electronic components are miniaturized electronic devices, including chip resistors, multilayer ceramic capacitors (MLCCs), and multilayer inductors. They are widely used in various instruments and electronic equipment due to their small size, low cost, and high performance [1,2]. Taping is the final process step; Figure 1 illustrates the taping process. First, operators manually load component pellets into a hopper, which feeds them into a vibrating bowl. The bowl arranges the components in an ordered manner through vibration, and a straight rail then transports them to an indexing plate. Electrical performance and appearance are two key indicators of chip component quality. Electrical performance can be characterized by electrical parameters such as capacitance, inductance, and resistance, whereas appearance can be quantitatively assessed using machine-vision methods [3]. Components sequentially pass through an electrical testing area and a visual defect inspection area on the indexing plate. Nonconforming items are automatically discharged through the corresponding NG (No Good) outlets, while qualified items are placed into the carrier tape cavities by a suction nozzle. A tape-collecting motor pulls the carrier tape forward. A missed-inspection device verifies whether a component is present in each cavity and performs a secondary appearance inspection. Finally, the cover tape is applied over the carrier tape under the action of an upper soldering iron and a pressure roller, and the components are sealed into reels according to the preset quantity.

The quality of chip electronic components can be described by electrical performance and appearance. Electrical performance is mainly affected by upstream manufacturing steps, whereas the taping process has a relatively minor influence. In contrast, appearance can be significantly affected by collisions and scratches during taping. Therefore, the “picture yield,” which characterizes appearance quality, is the quality indicator of interest in this paper; for convenience, the term “yield” hereafter refers to “picture yield”. Chip electronic components are manufactured in batches. When producing items within the same batch, key production factors (including personnel, machines, materials, process methods, environment, measurement, etc.) are relatively stable. The picture yield of a batch is defined as the ratio of the number of appearance-qualified items to the total number of items. In recent years, multiple chip component manufacturers have reported frequent low-yield batches in the taping process and substantial batch-to-batch fluctuations in picture yield. Investigating the specific factors that affect picture yield is therefore necessary, as it enables engineers to conduct targeted mistake-proofing and maintenance.

The internal structure of a taping machine is complex, involving many process stages with strong coupling. Moreover, numerous latent variables in the production factors (e.g., subtle differences between material lots, equipment state drift, environmental disturbances, and certain process details that are difficult to acquire online) make it challenging to establish a high-fidelity physics-based model that covers the entire process. In this context, virtual metrology (VM) monitors and fuses industrial process parameters and leverages machine learning (ML) to learn the mapping between key parameters and quality indicators, thereby enabling online quality assessment and prediction. VM has formed a relatively mature research and application ecosystem in semiconductor manufacturing, and has also been increasingly introduced into machining, metal processing, and chemical processing for quality management [4,5,6].

Recent semiconductor VM studies have advanced along several representative directions. For example, Xie et al. proposed a true sparse principal component analysis (PCA) method to reduce the number of essential sensors in VM while preserving the explanatory capability of the extracted components [7], and Wang et al. further developed a dynamic sparse PCA framework for sensor data dimensionality reduction with improved global sparsity across principal components [8]. Beyond sensor reduction, Chen et al. introduced a multimodal hierarchical learning architecture for semiconductor VM, in which samples are first divided into yield-related modes and then modeled through a classification–regression hierarchy [9]. Deivendran et al. studied VM for chemical mechanical planarization (CMP) of semiconductor wafers and demonstrated the effectiveness of data-driven modeling for process-quality estimation in a specific semiconductor process [10]. More recently, Wu et al. proposed an adaptive multi-view Bayesian co-training framework for semi-supervised VM, showing that unlabeled wafer data can be exploited to improve prediction performance when metrology labels are scarce [11]. These studies indicate that recent semiconductor VM research has mainly evolved around sensor/representation reduction, multimodal modeling, data-efficient learning, and process-specific quality estimation.

Recent VM research in other industrial domains has also shown continued methodological development. In machining, Tian et al. proposed a fuzzy echo state broad learning system for surface roughness VM, highlighting the value of dynamic learning architectures for capturing nonlinear and time-varying process characteristics [12]. In metal surface finishing and polishing, Yang et al. developed a combined virtual sample generation and feature construction model to improve small-sample surface roughness prediction, demonstrating the potential of data augmentation and feature enhancement for data-driven quality prediction [13]. In chemical processing, Han et al. introduced a semi-supervised robust learning framework for dynamic generative latent variable models and applied it to industrial VM, showing that robust latent variable modeling can effectively exploit unlabeled data and handle dynamic process uncertainty [14]. These studies suggest that, beyond semiconductor manufacturing, VM has been extended to a wider range of industrial scenarios, where dynamic learning, small-sample enhancement, and semi-supervised robust modeling have become important methodological themes.

After approximately two decades of development, VM has become an established data-driven approach for quality estimation across multiple manufacturing domains. However, related studies in electronic component manufacturing (e.g., MLCCs) remain limited, especially for in-line yield VM in actual production environments. Existing public research more often focuses on data-driven quality and reliability modeling for component-level assessment, experimental process optimization, or lifetime evolution [15], rather than on production-line-oriented yield prediction. For example, Li et al. developed a hybrid framework that combines physics-of-failure modeling with neural-network-based error compensation to predict MLCC reliability under thermal–electrical coupling operating conditions [16]. In a related but assembly-level setting, Yu et al. developed ML surrogate models trained on finite element simulation data to predict creep-strain-based reliability indicators of solder joints in multilayer chip capacitors under thermal cycling [17]; while these studies demonstrate the value of data-driven methods for reliability-oriented analysis of electronic components, they mainly target offline reliability evaluation under experimental or simulation-assisted conditions, rather than real-time yield prediction directly driven by production line process data. Consistent with the limited literature, our collaborative experience with several chip electronic component manufacturers also suggests that VM has not yet been deployed in their actual production lines at the time of this study. A key engineering difficulty is that manufacturing data in this scenario often exhibit incomplete features and strong label imbalance.

(1) Incomplete features and latent variable effects: The manufacturing process of chip electronic components spans multiple steps and equipment, and some key factors are difficult to acquire online or cannot be strictly aligned to individual samples. As a result, the training data exhibit partial observability: data from different steps/systems are inconsistent in variable coverage, and unobserved latent states (e.g., subtle differences between incoming material lots, equipment state drift, and environmental disturbances) may substantially affect yield. In the context of statistics and causal learning, this issue is often discussed in terms of omitted variable bias and identifiability challenges under partial observability. For this study, the more direct implication is that, under the currently observable variable set, the observed yield (Y) often contains considerable unexplained fluctuations driven by unobserved factors, making direct batch-wise yield prediction difficult to stabilize in practice. On the one hand, prior work has provided computable bounds and sensitivity-characterization approaches for estimation bias in the presence of omitted variables, addressing the extent to which unmeasured factors may induce systematic deviations [18]. On the other hand, under the reality of inconsistent variable coverage in multi-source data, studies have shown that whether the target relationship can be uniquely determined from the available observational distributions depends on the coverage and stitching conditions of the observed subsets with respect to the target quantity [19]. In addition, some work explicitly introduces latent variables into models to absorb the impact of unobserved factors, thereby improving estimation robustness [20]. These studies provide two direct insights for this work. First, unmeasured factors may introduce systematic deviations or unexplained variability at the global scale; therefore, a mechanism is needed to localize their influence, reduce model sensitivity to unobserved disturbances, and improve reproducible prediction consistency. Second, when data sources and variable coverage are inconsistent, approaches that rely on strict causal identification or latent variable structural assumptions typically require additional information (e.g., intervention data, reliable sets of proxy variables, or verifiable structural assumptions) and stronger statistical conditions. For the real industrial data considered here, it is currently difficult to obtain sufficient intervention/control information, and it is also difficult to impose testable structural specifications on the latent variable generation mechanism. Moreover, the goal of this paper is to build a deployable VM and yield-prediction model rather than to identify strict causal effects. Therefore, instead of introducing complex causal-identification or latent variable inference models, we focus on a data-driven and deployable stratification strategy, and redefine the modeling target as predicting the central tendency of yield under the same observable operating conditions, characterized by the within-bin median, i.e., the typical yield. Furthermore, for subsequent modeling and deployment, we organize the “binned operating condition units and their representative samples” into an operating condition dictionary: each operating condition unit corresponds to one representative sample and its typical yield label, forming a compact and reusable training set representation. Motivated by these insights and considering engineering practicality and deployability, we stratify the feature space under process-prior constraints via multidimensional equal-frequency binning. By constructing locally homogeneous sample subsets in the multidimensional space, global uncertainty induced by unobserved factors is transformed into stratified local fluctuations, thereby reducing the learning process’s sensitivity to omitted-variable disturbances and improving model stability and practical usability. Specifically, after multidimensional binning, we use the median of the observed yields within each operating condition unit to characterize the typical yield, and construct an operating condition dictionary composed of feature bin center values and typical yields. Compared with equal-width binning, we adopt equal-frequency binning because industrial data are often skewed and tail samples are scarce. Equal-width partitioning can easily produce many empty bins or extremely sparse regions, leading to unstable local statistics and model estimates, and may even amplify incidental fluctuations caused by sparsity. In contrast, equal-frequency binning uses quantile-based boundaries to make sample sizes across bins as balanced as possible, improving the statistical reliability of stratification under limited sample sizes and providing a more robust basis for subsequent within-stratum learning and sample weighting.

(2) Label imbalance and tail-oriented learning: Chip electronic component manufacturing typically aims at stable high-yield production, which results in a low proportion of low-yield samples; however, these samples carry the highest quality risk and engineering value. Consequently, conventional regression/classification models tend to fit the majority “normal/high-yield” region during training, while exhibiting insufficient accuracy and risk-discrimination capability in the low-yield tail. For imbalanced learning, prior studies have systematically summarized cost-sensitive learning, resampling, and semi-supervised/PU (Positive Unlabeled) approaches in classification tasks to address real-world data with scarce positives and uncertain labels [21]. In industrial fault diagnosis, strategies such as “label-sampling space augmentation” have also been proposed to improve recognition of rare classes [22]. More closely aligned with our setting is imbalanced regression, where the response variable is continuous and samples in critical regions are scarce. Related surveys indicate that resampling and cost-sensitive strategies can substantially improve learning in rare regions in imbalanced regression, but there is no universally optimal solution across all datasets and learners; designs must be tailored to the task objective and the underlying data distribution [23]. In addition, studies on imbalanced regression under distribution-biased data further suggest that introducing static and computable sample importance weights can mitigate distribution bias and enhance learning on critical samples with little additional training cost [24]. Based on these insights, we incorporate a sample-weighting mechanism in both modeling and evaluation, assigning higher importance to low-yield samples so that the optimization objective places greater emphasis on prediction performance in the low-yield region and on high-risk samples. We couple the weighting strategy with the aforementioned stratification results from multidimensional equal-frequency binning to avoid overly amplifying nuisance factors or inducing local overfitting that can arise from naive threshold-based weighting, thereby ensuring consistency between the weighting scheme and the data structure.

In industrial settings, training samples are often limited and data distributions can drift across batches and over time, so care is needed when estimating generalization performance after model selection. Varma and Simon showed that using cross-validation for model selection can bias error estimation and lead to optimistic performance reporting [25]. Cawley and Talbot further analyzed overfitting in model selection and the resulting selection bias in performance evaluation [26]. Wainer and Cawley revisited nested cross-validation and discussed its practical role and limitations in obtaining less biased performance assessment under repeated comparisons [27]. Accordingly, we adopt a nested cross-validation (NCV) framework, where the outer loop is used for algorithm comparison and the inner loop combines Bayesian optimization (BO) for hyperparameter search.

Importantly, due to the limited sample size, the binning boundaries and the operating condition dictionary in this study are constructed once using the full dataset and are not rebuilt within each fold. Therefore, the outer-fold results should be interpreted as conditional performance under a fixed operating condition dictionary rather than strict deployment-time generalization to completely unseen future data. In a more deployment-aligned setting, the dictionary can be constructed on a historical data window, future batches can be mapped to the predefined bins for online prediction, and the dictionary/model can then be rebuilt or incrementally updated as new production data accumulate. We have not yet implemented such a temporal online simulation evaluation within the outer folds mainly because the current dataset has limited time span and sequential coverage, and reliable assessment of drift monitoring and updating strategies typically requires larger-scale continuous production data. Accordingly, the conditional performance reported in this paper should be regarded as a relatively optimistic reference for future deployment-oriented evaluation.

In summary, this study addresses an underexplored VM setting: batch-level picture yield prediction for electronic component taping under partial observability. Rather than proposing a new standalone regressor, the contribution lies in a problem-specific integrated formulation tailored to this data regime, built around target reformulation, operating condition dictionary construction, and tail-oriented learning under a NCV framework. This formulation is intended to improve modelability and evaluation stability under the practical constraints of the available industrial data. This positioning distinguishes the present work in two respects. First, compared with prior VM studies, it addresses a different problem setting, namely in-line yield prediction under partial observability, incomplete features, and sparse low-yield samples. Second, compared with existing data-driven studies on electronic components, it targets a different prediction object: production line picture yield derived from real manufacturing process data, rather than offline reliability, lifetime evolution, or experimentally or simulation-assisted quality indicators.

**Figure 2:**
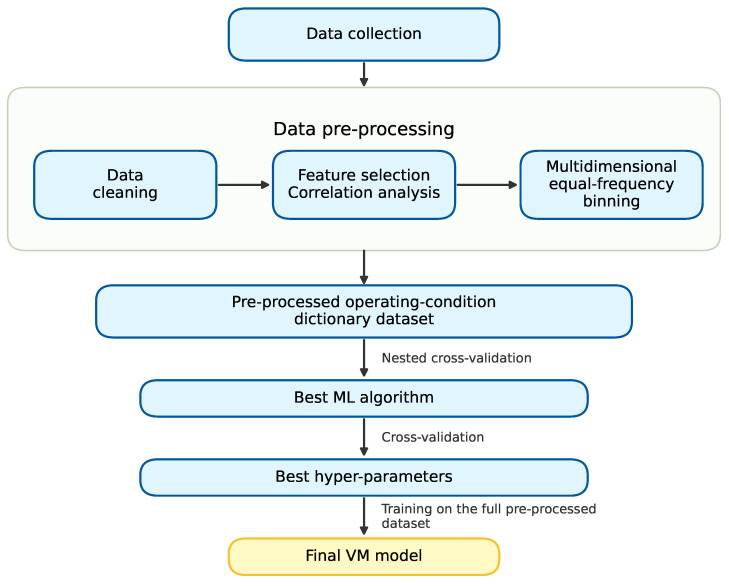
Workflow of the proposed VM modeling framework based on operating condition dictionary.

The general procedure used in this paper is illustrated in Figure 2. In the data preprocessing stage, we first clean the collected data, extract key features according to the process flow, and perform correlation analyses between the key features and yield using multiple methods. We then apply multidimensional equal-frequency binning to construct the operating condition dictionary dataset. Using this preprocessed dataset and multiple ML algorithms, we perform NCV to compare candidate learners. Within the inner loop, BO is used for hyperparameter tuning. Finally, using the selected algorithm and its best hyperparameter configuration, we train the final typical yield model on the full preprocessed dataset. Higher weights are assigned to low-yield samples during model training.

**The main contributions of this paper are as follows:**

1. We extend VM to an underexplored industrial scenario, namely production-line-oriented yield prediction for electronic component taping, using real desensitized manufacturing data.
2. We propose an operating condition dictionary representation based on multidimensional equal-frequency binning and within-bin median typical yield, which improves data modelability under incomplete features and unobserved disturbances.
3. We establish an NCV+BO-based conditional evaluation framework under a fixed operating condition dictionary and integrate sample weighting for low-yield-oriented learning.

The remainder of this paper is organized as follows. Section 2 presents an overview of the dataset and the pre-modeling workflow, including data acquisition, data cleaning, feature extraction and selection, and multidimensional equal-frequency binning. Section 3 introduces the ML algorithm selection and hyperparameter tuning framework based on NCV and BO. Section 4 reports the modeling results of multiple algorithms under this framework. Section 5 discusses the effects of multidimensional equal-frequency binning, sample weighting, and different hyperparameter-optimization strategies through ablation studies, and analyzes the limitations of the proposed method. Finally, Section 6 concludes the paper and outlines future work.

## 2. Dataset and Preprocessing

### 2.1. Data Collection and Cleaning

The analysis data were collected using an in-house data-acquisition system developed by Guangdong Electronic Technology Research Institute. When the acquisition program starts, it retrieves, via the connection addresses maintained in configuration files, the industry–PC interfacing method and data templates from the acquisition server, and then locates the corresponding files or databases for data reading. After obtaining the data, it performs field mapping according to the data-template settings, and finally transmits the mapped data to the acquisition server, where the acquisition program uploads them to the database.

The data ultimately uploaded to the database include taping machine equipment parameters, error/alarm parameters, the number of qualified items, and the number of appearance-nonconforming items. Parameters that describe production factors are referred to as features. During data export, the collected data are automatically desensitized. Specifically, each feature undergoes a linear transformation. For the *i*-th sample of feature *x*, the value before desensitization is $x_{i}$, and the value after desensitization is $x_{i,scale}$:

(1) $$x_{i,scale}=\alpha x_{i},$$

where α is a positive random number automatically generated by the system (for each exported batch, an α is generated for each feature), and α is not recorded. The desensitization procedure does not change the relative ordering of sample values within each feature; therefore, its impact on subsequent correlation analysis, independence testing, and VM model training can be neglected. The desensitized data used in this study span approximately two months and contain about 10,000 batches.

The raw data are noisy and unstructured, and thus require data cleaning. Data cleaning is performed at the batch level, including yield computation, format conversion, missing value detection, deduplication, removal of ambiguous records, and data integration. First, yield is computed using the number of items discharged at the picture NG outlet and the number of items discharged at the qualified product outlet shown in Figure 1. Next, through the steps from format conversion to data integration, the data are transformed into a format in which features and yield are aligned one-to-one. Each sample is an (N+1)-dimensional vector, where *N* is the number of features and the additional dimension corresponds to the label (yield). Thus, each sample summarizes the information of one production batch.

**Figure 3:**
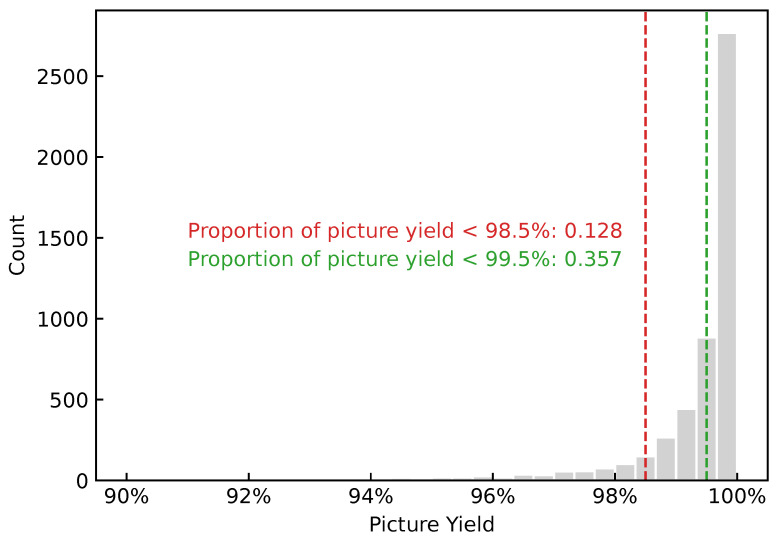
Distribution of picture yield after the data cleaning for a total of 5209 taping batch samples.

After data cleaning, a total of 5209 taping batch samples were obtained, as shown in Figure 3. Yield control in electronic component manufacturing is extremely strict. In general, batches with yield lower than 98.5% are considered unqualified, and batches with yield lower than 99.5% are considered low-yield. As can be seen from Figure 3, unqualified and low-yield batches account for 0.128 and 0.357, respectively; therefore, investigating the factors associated with low yield in these batches is necessary.

### 2.2. Feature Selection and Correlation Analysis

As described above, the product yield is computed from the number of items discharged at the picture NG outlet and the number of items discharged at the qualified product outlet shown in Figure 1. Therefore, feature parameters associated with process steps downstream of the qualified product outlet (e.g., material properties of the carrier tape, cover tape, and bottom tape) cannot affect the appearance inspection result from a physical-mechanism perspective. Based on this process constraint, such features are not considered in this study.

After removing variables that have no direct physical relevance to yield, we finally retain seven feature parameters related to appearance inspection and its upstream process steps; these features correspond to various equipment error/alarm counts. To systematically analyze the statistical relationships between these features and yield, we investigate them from three perspectives: linear correlation, monotonic correlation, and nonlinear statistical dependence.

First, the Pearson correlation coefficient [28] is used to assess the linear correlation between each feature and yield. The formula is given as follows:

(2) $$\rho \left(X;Y\right)=\frac{\sum_{i=1}^{s}\left(x_{i}-\bar{x}\right)\left(y_{i}-\bar{y}\right)}{\sqrt{\sum_{i=1}^{s}{\left(x_{i}-\bar{x}\right)}^{2}\sum_{i=1}^{s}{\left(y_{i}-\bar{y}\right)}^{2}}},$$

where *X* denotes a feature and *Y* denotes the yield. $x_{i}$ and $y_{i}$ denote the sample values of *X* and *Y*, respectively, $\bar{x}$, $\bar{y}$ denote the sample means, and *s* is the total number of samples. The Pearson correlation coefficient measures the strength of a linear relationship between two variables.

Considering that relationships in manufacturing process data are often nonlinear, we further employ the Spearman rank correlation coefficient [29] to examine whether a monotonic relationship exists between each feature and yield. It is defined as follows:

(3) $$\rho_{s}\left(X;Y\right)=1-\frac{6\sum_{i=1}^{s}d_{i}^{2}}{s(s^{2}-1)},$$

where $d_{i}$ is the rank difference between the feature variable and the yield at the *i*-th sample. The Spearman correlation coefficient is based on rank information and can capture monotonic but potentially nonlinear statistical relationships.

However, both correlation coefficients above are limited in their ability to reflect complex nonlinear and non-monotonic dependencies. Therefore, we further introduce mutual information as a supplementary criterion for feature selection.

Mutual information can quantify statistical dependence between two variables in an arbitrary form. In this study, we compute mutual information for continuous variables using a nonparametric *k*-nearest-neighbor estimator. Specifically, based on the Kraskov–Stögbauer–Grassberger (KSG) mutual information estimator [30], for a given set of *s* sample points ${\left\{\left(x_{i},y_{i}\right)\right\}}_{i=1}^{s}$, we first apply min–max normalization to the feature values $x_{i}$. The mutual information can then be estimated as follows:

(4) $$I\left(X;Y\right)=\psi \left(k\right)-\frac{1}{s}\sum_{i=1}^{s}\left[\psi \left(n_{x}\left(i\right)+1\right)+\psi \left(n_{y}\left(i\right)+1\right)\right]+\psi \left(s\right),$$

where ψ(·) denotes the digamma function and *k* is the number of nearest neighbors. In this study, *k* is set to a small integer that is commonly used in practice to balance estimation bias and variance; within this range, the mutual information estimates are not sensitive to the choice of *k*. The quantities $n_{x}\left(i\right)$ and $n_{y}\left(i\right)$ denote, at the *i*-th sample, the numbers of points in the marginal *x*-space and *y*-space, respectively, that fall within a radius equal to the distance to the *k*-th nearest neighbor in the joint space [30]. This approach does not require discretization of continuous variables and can provide stable estimates of nonlinear statistical dependence under finite-sample settings.

Table 1 summarizes the Pearson correlation coefficients, Spearman correlation coefficients, and mutual information results between the seven features and yield.

As shown in Table 1, the Pearson correlation coefficients between the features and yield are generally small, indicating weak negative linear correlations. The Spearman correlation coefficients are stronger for some features, but they are still within a weak-correlation range overall, suggesting that only weak monotonic relationships exist between the variables. The mutual information results indicate that all features exhibit nonzero statistical dependence with yield.

These findings suggest that the relationships between yield and process features are complex and nonlinear. Moreover, the rankings obtained by sorting the three measures are not consistent. Relying on a single criterion for feature screening may therefore discard useful information. Given that the number of extracted features is small, we retain all seven features for subsequent modeling and adopt models capable of capturing nonlinear relationships for prediction and analysis.

### 2.3. Multidimensional Equal-Frequency Binning Strategy

The correlation analysis above indicates that it is difficult to establish a stable global mapping from any single feature to yield. This does not imply that the features are uninformative; rather, it reflects that yield depends on multidimensional feature combinations and their conditional distributions. Based on this observation, we further adopt multidimensional equal-frequency binning to reduce the model’s sensitivity to disturbance factors from the perspective of feature combinations.

In practical industrial production, factors that lead to batch-to-batch yield fluctuations can be divided into two categories. The first category consists of production elements, including personnel, machines, materials, process methods, and environment. Some of these elements can be captured by the data-acquisition system and thus extracted as features, whereas others cannot be captured. For example, during appearance inspection, clamps are used to fix the products in place, but the actual stress applied to the products cannot be accurately measured. The second category comprises random factors. As shown in Figure 1, components may collide with each other during transportation from the hopper to the indexing plate. For batches with a small number of items, such random collisions may induce substantial yield fluctuations for the entire batch. We collectively refer to the unmeasured production elements and random factors as disturbance factors.

**Figure 4:**
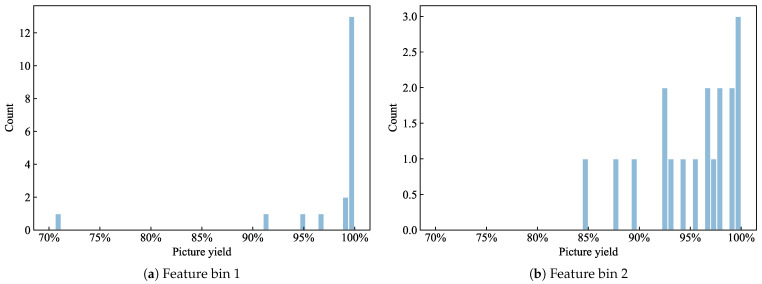
Yield distributions within two different feature bins.

Figure 4 shows the picture yield distributions in two different feature bins. Because samples within the same feature bin have similar feature values, the within-bin yield fluctuations can be approximately attributed to disturbance factors, whereas the differences between bins are mainly driven by differences in observable feature combinations. Based on this view, we do not treat the observed yield of an individual batch as a fully predictable target. Instead, after binning we define the typical yield as the central tendency under the same feature combination. Specifically, for each feature bin, the median of the observed yields within the bin is used as an empirical estimate of the yield trend under that operating condition, and an operating condition dictionary is constructed from the feature bin center values and the typical yields. In this way, without introducing additional prior assumptions, the typical yield level under each feature combination is retained while the influence of extreme values on training is attenuated.

**Figure 5:**
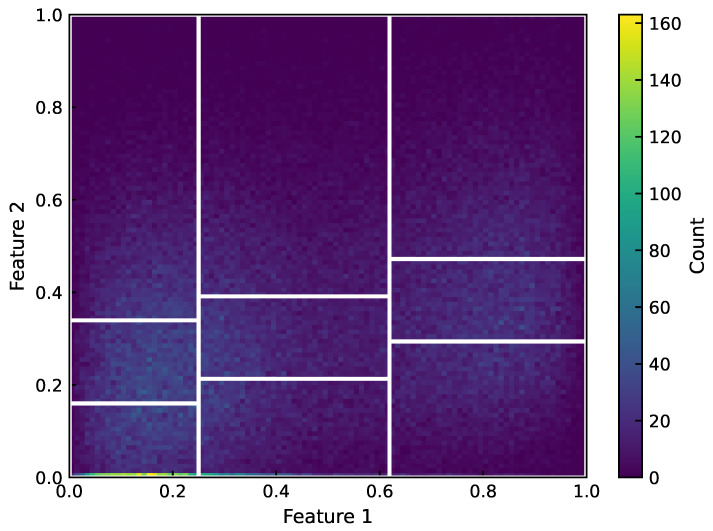
Sketch of conditional binning strategy for 2-dimensional case, the binning boundaries were overlaid on a fine-grained equal-width 2D histogram for visualization. The figure is generated with fake data for explanation.

In terms of specific technical implementation, we adopt a conditional binning strategy. First, the marginal quantiles of the first feature are used to partition it into *m* equal-frequency bins. Within each f0 bin, we further partition f1 into *n* equal-frequency bins using conditional quantiles, thereby forming an m×n grid structure such that the number of samples in each grid cell is approximately equal. The two-dimensional case is illustrated in Figure 5. For higher-dimensional settings, this strategy can be extended in the same manner. This recursive binning procedure guarantees that, for the data used in this study, the sample counts across all feature bins differ by at most 1. We performed sensitivity checks on several binning orders and did not observe any substantive changes in the conclusions. In this paper, the binning order is determined according to the temporal order of features along the process flow (i.e., a larger feature index indicates a later position in the process flow). The number of bins for each feature is listed in Table 2. The binning configuration is determined based on the statistical analysis in Section 2.2. In particular, f2 and f6 exhibit the strongest statistical dependence with yield in terms of mutual information, and their Pearson and Spearman correlations also have relatively large absolute values. Therefore, we use a finer binning granularity for f2 and f6 (three bins for each) to better capture key nonlinear variation regimes, whereas the remaining features use a coarser granularity to balance representational capacity against the risk of sparsity. The impact of the binning strategy on the results is discussed in Section 5.

Evaluation protocol note. In this study, the equal-frequency bin boundaries (quantile thresholds) are estimated once from the complete cleaned dataset and then kept fixed. The resulting operating condition dictionary (288 representative entries) is used as the modeling dataset in the subsequent NCV procedure, i.e., the binning/dictionary is not reconstructed within each cross-validation fold.

Formally, let the observed yield of the *i*-th batch be $y_{i}\in \left[0,1\right]$, the feature vector be $x_{i}$, and the binning mapping be $g(x_{i})$. For any bin *b*, denote its index set by $I_{b}=\left\{i\;|\;g\left(x_{i}\right)=b\right\}$, and its sample size by $N_{b}=\left|I_{b}\right|$. We treat samples ${\left\{\left(x_{i},y_{i}\right)\right\}}_{i\in I_{b}}$ within the same bin as approximately independent and identically distributed (i.i.d.) observations conditional on the operating condition unit *b*. Accordingly, the typical yield for bin *b* is defined as the median of the observed yields within the bin:

(5) $$y_{b}^{★}=\mathrm{median}\left\{y_{i}\;|\;i\in I_{b}\right\}.$$

On the other hand, to obtain a representative feature vector that corresponds uniquely to operating condition unit *b*, we use the center of the bin interval in feature space as its prototype representation. Specifically, let the bin interval of unit *b* on the *j*-th feature dimension be $\left[l_{b,j},u_{b,j}\right)$ (determined by equal-frequency quantile thresholds; the last interval is $\left[l_{b,j},u_{b,j}\right]$). We then define

(6) $$x_{i_{b}}=c_{b}=\left(\frac{l_{b,1}+u_{b,1}}{2},\frac{l_{b,2}+u_{b,2}}{2},\ldots ,\frac{l_{b,d}+u_{b,d}}{2}\right).$$

This yields the operating condition dictionary entry $\left(x_{i_{b}},y_{b}^{★}\right)$. In subsequent modeling, the typical yield $y^{★}$ is used as the supervised label. When the context is clear, the superscript in $y^{★}$ may be omitted and *y* is used to denote the typical yield. After multidimensional equal-frequency binning, each operating condition unit is represented by one center prototype, resulting in 288 operating condition dictionary samples. Although the sample size is substantially reduced, each sample corresponds to a representative state of an operating condition unit, forming a more compact training set representation, reducing redundancy in high-density regions, and aligning more closely with the task definition of typical yield.

**Figure 6:**
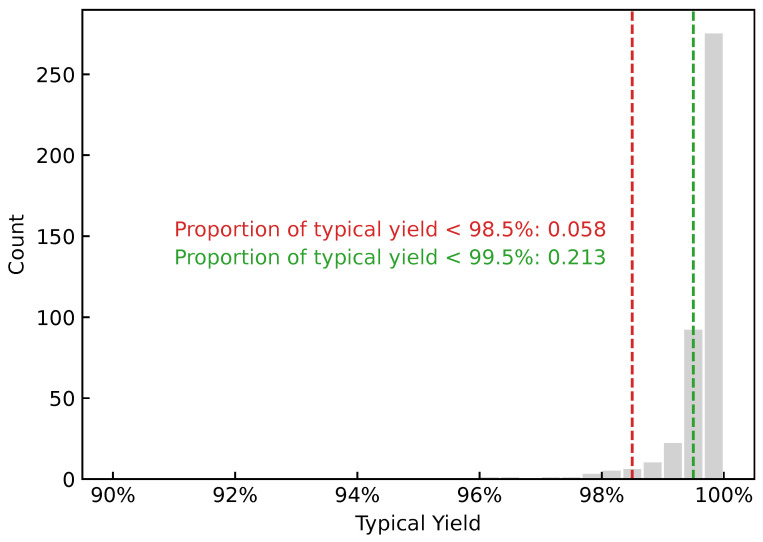
Distribution of typical yields after multidimensional equal-frequency binning.

After multidimensional equal-frequency binning, the distribution of typical yields is shown in Figure 6. Compared with the batch-level yield distribution (Figure 3), the numbers of unqualified (<98.5%) and low-yield (<99.5%) samples in the typical yield distribution are substantially reduced. This is because the within-bin median is used as the typical yield after binning, and extremely low-yield samples are often absorbed by the median. This target reformulation reduces label variance and changes the difficulty of the learning task; its impact is later examined through the ablation study in Section 5.

### 2.4. Summary of Section 2

In this section, we constructed and preprocessed the taping-process dataset. After desensitization and data cleaning, the raw data were organized into batch-level “feature–yield” samples. In total, 5209 valid batches were obtained, and the yield distribution is characterized by a dominant high-yield region with relatively scarce low-yield samples.

Building on this dataset, we first conducted a process-mechanism-based screening of features and retained seven key features associated with appearance inspection and its upstream processes. We then used the Pearson correlation coefficient, Spearman rank correlation coefficient, and the KSG-based mutual information estimator to characterize the statistical relationships between features and yield from three perspectives: linear correlation, monotonic association, and nonlinear dependence. The results indicate that each single feature is only weakly correlated with yield overall, yet exhibits nonzero statistical dependence, suggesting that yield is more likely determined by multidimensional feature combinations and their conditional distributions.

To address batch-to-batch differences caused by unobserved factors and random fluctuations, we further proposed a multidimensional equal-frequency binning strategy that discretizes the seven-dimensional observable feature space into a finite set of operating condition units (feature bins). For each operating condition unit, we summarize the observed picture yield distribution of samples in the bin and define the typical yield of that unit as the within-bin median, denoted by $y_{b}^{*}$. We represent each unit by the center vector of its bin intervals, $c_{b}$, thereby constructing an “operating condition dictionary” composed of $\left\{\left(c_{b},\;y_{b}^{*}\right)\right\}$. The binning granularity is determined based on the statistical analysis: finer bins are assigned to key features with stronger dependence to balance operating-condition expressiveness against sufficient sample support within each partition. As a result, 288 operating condition dictionary entries are obtained, providing a more structured and modelable data foundation for subsequent VM modeling to estimate typical yields at the operating-condition level and to perform pre-screening for new batches.

## 3. NCV and BO Framework

After multidimensional equal-frequency binning, to obtain a predictive model for typical yield under disturbance factors and a skewed target distribution (where high-yield samples dominate and low-yield samples are scarce), we construct a unified modeling and tuning framework that integrates NCV [31] and BO [32]. To prevent model training from being dominated by high-yield samples and to strengthen learning on low-yield risk samples, we introduce a sample-weighting mechanism: by setting a yield threshold and assigning a higher loss contribution to the low-yield region, low-yield samples receive larger weights in the optimization objective, thereby improving the model’s fitting and discrimination capability in critical risk regions. Under this framework, hyperparameters are selected via the inner cross-validation loop, the best-performing ML algorithm is chosen via the outer cross-validation loop, and the final model is trained on the full dataset of typical yields.

### 3.1. Machine Learning Algorithm Selection

The task in this study is essentially supervised regression: predicting yield using multi-source device-operation features and error/alarm statistics. Considering that industrial tabular data often exhibit nonlinear relationships, strong feature correlations, and the coexistence of outliers and distribution drift, we compare four representative regression models: a feedforward neural network (FNN), XGBoost (XGB), random forest (RF), and support vector regression (SVR) [33]. These models cover different function-approximation mechanisms and bias–variance trade-offs, providing complementary baselines under confidential data and limited-sample settings, thereby improving the robustness and reproducibility of the conclusions.

- FNN [34]: Also known as a multilayer perceptron (MLP), it learns complex feature interactions through multilayer nonlinear mappings and is suitable for end-to-end modeling with multi-source fused features. It is used to examine whether the data contain higher-order nonlinear structures that require stronger expressive capacity to capture.
- XGB [35]: An efficient implementation of gradient-boosted trees, it is well suited for modeling nonlinearities and feature interactions in tabular data and often performs stably in industrial scenarios. It serves as a strong baseline to achieve competitive predictive accuracy.
- RF [36]: A tree ensemble based on bootstrap sampling and random feature subsets, offering strong robustness and variance reduction. It is used as a robust baseline to compare stability differences between boosted trees and neural networks under outliers/disturbance factors.
- SVR [37]: A maximum-margin regression method based on the kernel trick, which is often competitive under small-to-medium sample sizes. As a kernel-method baseline with a mechanism different from tree models and neural networks, it is used to assess whether the task can be effectively characterized by a relatively smooth nonlinear mapping.

### 3.2. Nested Cross-Validation

#### 3.2.1. Problem Definition and Notation

Let the operating condition dictionary dataset be $D={\left\{\left(x_{i},y_{i}\right)\right\}}_{i=1}^{N}$, where $x_{i}\in R^{d}$ denotes the center value of a feature bin, and $y_{i}\in \left[0,1\right]$ denotes the typical yield, as defined in Section 2.3.

Because samples with high typical yield dominate in industrial settings, directly regressing *y* can lead to insufficient fitting in the low-yield risk region. To enhance discrimination in the low-yield (high-defect) region, we transform the learning target to the log domain of the defect rate:

(7) $$d_{i}=1-y_{i},\;t_{i}=\log\left(d_{i}+\epsilon_{d}\right),$$

where $d_{i}\in \left[0,1\right]$ is the defect rate and $\epsilon_{d}>0$ is a stabilization constant to avoid log(0). In this study, we set $\epsilon_{d}=10^{-8}$. During training and hyperparameter tuning, the model consistently uses *t* as the regression target, which improves numerical resolution in the high-yield region where y≈1 and increases error sensitivity to the low-yield tail.

Given a learning algorithm family A and its hyperparameter space Θ, the training process can be written as

(8) $$\hat{f}=A\left(D_{\mathrm{train}};\theta \right),\;\theta \in \Theta ,$$

where $\hat{f}:x\mapsto \hat{t}$ is the regressor fitted on the training folds.

During prediction, after the model outputs $\hat{t}$, we apply the inverse transformation to obtain the predicted defect rate and yield:

(9) $$\tilde{d}=\exp\left(\hat{t}\right)-\epsilon_{d},\;\tilde{y}=1-\tilde{d}.$$

Because the regressor may produce out-of-range values numerically (e.g., $\tilde{d}<0$ or $\tilde{d}>1$), which leads to $\tilde{y}\notin \left[0,1\right]$, we impose physical boundary constraints on the outputs. For all model outputs (including FNN, SVR, RF, and XGB), we consistently apply a post-processing projection to clip predictions into [0,1]:

(10) $$\hat{y}=\Pi_{\left[0,1\right]}\left(\tilde{y}\right),\;\Pi_{\left[0,1\right]}\left(u\right)=\min\left\{1,\max\left\{0,u\right\}\right\}.$$

Here, $\hat{y}$ is the final predicted typical yield used for outer-loop performance evaluation.

In our implementation, we apply feature transformations that match the model characteristics. For FNN and SVR, we first apply a log(1+x) transformation to compress long-tailed distributions, followed by min–max normalization. For RF and XGB [35], no normalization is performed. This is because tree-based models mainly rely on relative ordering and threshold splits of features and are insensitive to linear scale transformations; therefore, normalization is omitted to reduce redundant preprocessing and fold-specific fitting steps. Let the *p*-th feature dimension be $x^{\left(p\right)}$. For FNN/SVR, we use

(11) $$x^{\left(p\right)}\leftarrow \log\;\left(1+x^{\left(p\right)}\right),\;x^{\left(p\right)}\leftarrow \frac{x^{\left(p\right)}-\min\left(x^{\left(p\right)}\right)}{\max\left(x^{\left(p\right)}\right)-\min\left(x^{\left(p\right)}\right)+\epsilon_{x}},$$

where $\epsilon_{x}$ is a stabilization constant.

Except for the binning boundaries and the operating condition dictionary in Section 2.3, all other preprocessing steps follow the principle of fitting parameters on the training folds and applying them to the validation/test folds. For example, feature-scaling parameters based on min(·) and max(·) are estimated without using any information from the validation/test folds.

We report multi-metric performance on the outer test folds in terms of the typical yield *y*, including mean squared error (MSE), weighted mean squared error (WMSE), mean squared error in the low-yield region (MSE(y<τ)), the coefficient of determination ($R^{2}$), and relative-error threshold accuracy (Acc(α))

(12) $$\begin{array}{cc} \mathrm{MSE} & =\frac{1}{M}\sum_{j=1}^{M}{\left({\hat{y}}_{j}-y_{j}\right)}^{2}, \end{array}$$

(13) $$\begin{array}{cc} \mathrm{WMSE} & =\frac{\sum_{j=1}^{M}w_{j}{\left({\hat{y}}_{j}-y_{j}\right)}^{2}}{\sum_{j=1}^{M}w_{j}}, \end{array}$$

(14) $$\begin{array}{cc} \mathrm{MSE}(y<\tau ) & =\frac{1}{M_{-}}\sum_{j\in J_{-}}{\left({\hat{y}}_{j}-y_{j}\right)}^{2}, \end{array}$$

(15) $$\begin{array}{cc} R^{2} & =1-\frac{\sum_{j=1}^{M}{\left({\hat{y}}_{j}-y_{j}\right)}^{2}}{\sum_{j=1}^{M}{\left(y_{j}-\bar{y}\right)}^{2}}, \end{array}$$

(16) $$\begin{array}{cc} \mathrm{Acc}(\alpha ) & =\frac{1}{M}\sum_{j=1}^{M}I\left(\frac{|{\hat{y}}_{j}-y_{j}|}{|y_{j}|+\epsilon }\leq \alpha \right), \end{array}$$

where *M* is the number of samples in the test fold, $\bar{y}$ is the mean of the ground-truth values in the test fold, and I(·) is the indicator function. The constant ϵ is a stabilization term in the denominator, and we set $\epsilon =10^{-8}$. The number of low-yield samples, denoted by $M_{-}$, is defined as

(17) $$J_{-}=\left\{j:y_{j}<\tau \right\},\;M_{-}=\left|J_{-}\right|.$$

We set the low-yield threshold to τ=0.995. We do not further focus on unqualified samples with $y_{j}<98.5\%$ because the number of unqualified samples in the typical yield data is extremely small (only 0.058 in proportion; see Figure 6), which may distort statistical results. In contrast, low-yield samples with $y_{j}<99.5\%$ account for 0.213, and even after up-weighting (total tail weight = 0.5), the amplified error does not dominate the overall objective.

When sample weighting is disabled, one can set $w_{j}\equiv 1$, in which case WMSE=MSE. The parameter α in Acc(α) can be set to 0.5%, 0.2%, etc., to meet engineering accuracy requirements.

#### 3.2.2. Structure and Motivation of NCV

When traditional *k*-fold cross-validation is used for hyperparameter selection, different configurations are repeatedly compared on the same validation splits. As a result, the selected “best” model can be inadvertently tuned to the validation data, leading to overly optimistic performance estimates. NCV mitigates this bias through a two-level structure with “outer evaluation” and “inner selection”:

- Outer loop: D is partitioned into $K_{\mathrm{out}}$ mutually exclusive folds. Each fold is in turn used as the outer test set $D_{\mathrm{test}}^{\left(k\right)}$, and the remaining folds form the outer training set $D_{\mathrm{train}}^{\left(k\right)}$.
- Inner loop: Within each outer training set $D_{\mathrm{train}}^{\left(k\right)}$, the data are further split into $K_{\mathrm{in}}$ inner folds for hyperparameter search, yielding the optimal hyperparameter configuration $\theta^{*(k)}$.
- Evaluation: The model is retrained on $D_{\mathrm{train}}^{\left(k\right)}$ using the configuration selected by the inner loop, and outer-loop performance metrics are computed on $D_{\mathrm{test}}^{\left(k\right)}$. Averaging over $k=1,\ldots ,K_{\mathrm{out}}$ provides an overall performance estimate, which is used for evaluating and selecting ML algorithms.

In our experiments, both the outer and inner numbers of folds are set to 5, i.e., $K_{\mathrm{out}}=5$ and $K_{\mathrm{in}}=5$. This setting provides stable evaluation while keeping the computational cost within an acceptable range.

#### 3.2.3. NCV Procedure

The inner loop aims to minimize the validation loss. For the *k*-th outer fold, let the outer training set $D_{\mathrm{train}}^{\left(k\right)}$ be partitioned into $K_{\mathrm{in}}$ mutually exclusive inner folds:

(18) $$D_{\mathrm{train}}^{\left(k\right)}=\overset{K_{\mathrm{in}}}{\underset{\ell =1}{⋃}}D^{\left(k,\ell \right)},\;D^{\left(k,\ell \right)}\cap D^{\left(k,m\right)}=⌀\;\left(\ell \neq m\right).$$

In the *ℓ*-th inner validation, the *ℓ*-th fold is used as the validation set and the union of the remaining folds is used as the training set, i.e.,

(19) $$D_{\mathrm{val}}^{\left(k,\ell \right)}=D^{\left(k,\ell \right)},\;D_{\mathrm{train}}^{\left(k,\ell \right)}=\overset{K_{\mathrm{in}}}{\underset{\begin{array}{c} m=1\\ m\neq \ell \end{array}}{⋃}}D^{\left(k,m\right)}.$$

Let the inner-loop validation loss function be L(·). Because the training target in this study is $t=\log(1-y+\epsilon_{d})$, inner-loop tuning is conducted based on errors in the *t* domain. To place more emphasis on low-yield samples, we adopt a fixed sample-weighting strategy (Section 3.2.4); therefore, the inner-loop loss is consistently set to the weighted mean squared error ${\mathrm{WMSE}}_{t}$. Here, ${\mathrm{WMSE}}_{t}$ denotes the weighted mean squared error computed after transforming the yield labels to the *t* domain, following the definition in Equation (13). For the *k*-th outer fold, the optimal hyperparameters obtained in the inner loop satisfy

(20) $$\theta^{*(k)}=\arg\min_{\theta \in \Theta }\frac{1}{K_{\mathrm{in}}}\sum_{\ell =1}^{K_{\mathrm{in}}}{\mathrm{WMSE}}_{t}\left(A\left(D_{\mathrm{train}}^{\left(k,\ell \right)};\theta \right),D_{\mathrm{val}}^{\left(k,\ell \right)}\right).$$

After determining $\theta^{*(k)}$, we retrain the model on $D_{\mathrm{train}}^{\left(k\right)}$ to obtain outer-fold predictions $\hat{t}$, and then apply the inverse transformation and boundary constraints to obtain $\hat{y}$. Finally, we report MSE, WMSE, MSE(y<τ), $R^{2}$, and Acc(α) on $D_{\mathrm{test}}^{\left(k\right)}$ based on *y*. The overall outer-loop performance is computed as the average across folds:

(21) $${\hat{E}}_{\mathrm{gen}}=\frac{1}{K_{\mathrm{out}}}\sum_{k=1}^{K_{\mathrm{out}}}{\mathrm{MSE}}^{\left(k\right)}\left(y,\hat{y}\right),$$

and we also report the mean and variance of ${\mathrm{WMSE}}^{\left(k\right)}\left(y,\hat{y}\right)$, ${\mathrm{MSE}}^{\left(k\right)}\left(y<\tau \right)$, $R^{2,(k)}$, and $\mathrm{Acc}{\left(\alpha \right)}^{\left(k\right)}$ to characterize the overall error and the tail-sensitive error performance on low-yield samples, respectively.

#### 3.2.4. Sample Weighting and Imbalance Handling

Industrial yield data typically exhibit a pronounced distribution skew: most samples concentrate in the high-yield region, whereas low-yield samples constitute a small proportion but carry higher engineering value. To prevent the training process from being dominated by high-yield samples and to explicitly strengthen learning on low-yield risk samples, we adopt a fixed and interpretable group-ratio weighting strategy. In each model fitting, this strategy enforces a controllable and higher relative contribution of “low-yield samples” (y<τ) versus “the remaining samples” (y≥τ) in the weighted loss. To keep consistency with the evaluation protocol (Section 3.2.1), we fix the threshold as

(22) τ=0.995.

For any data split (training or validation fold), we define two index sets:

(23) $$I_{-}=\left\{i:y_{i}<\tau \right\},\;I_{+}=\left\{i:y_{i}\geq \tau \right\},$$

and denote the sample counts by $n_{-}=\left|I_{-}\right|$ and $n_{+}=\left|I_{+}\right|$. To ensure that the low-yield group receives sufficient loss contribution even when it is scarce, we assign a constant weight within each group and enforce equal total weights for the two groups (thereby preventing low-yield samples from being overwhelmed by the numerically dominant high-yield samples):

(24) $$w_{i}=\left\{\begin{array}{cc} \rho , & i\in I_{-},\\ 1, & i\in I_{+}, \end{array}\right.\;\rho =\frac{n_{+}}{n_{-}}\;\left(n_{-}>0\right).$$

It follows that $\sum_{i\in I_{-}}w_{i}=\sum_{i\in I_{+}}w_{i}=n_{+}$, so that, in the weighted loss, the low-yield group and the remaining-sample group have overall influences of the same order of magnitude.

In the log-defect-rate domain *t*, we use the weighted mean squared error as the unified criterion for training and inner-loop validation:

(25) $$\frac{\sum_{j=1}^{M}w_{j}{\left({\hat{t}}_{j}-t_{j}\right)}^{2}}{\sum_{j=1}^{M}w_{j}},$$

where *M* is the number of samples in the current data split. This form preserves a consistent loss scale while allowing the weights to act as direct amplification factors on the squared-error terms, thereby imposing stronger penalties on errors for low-yield samples.

Both WMSE and MSE(y<τ) on the outer test folds are computed using the same threshold τ=0.995 by defining the corresponding sample sets within each test fold (for evaluation only and not used in training), as in Equation (13). For outer-fold WMSE, the weights $w_{i}$ are also computed using the test fold’s own $n_{-}$ and $n_{+}$ to ensure that the evaluation protocol is consistent with the group-weighting strategy used during training.

### 3.3. Bayesian Optimization

#### 3.3.1. Hyperparameter Optimization Problem

For a given algorithm A, hyperparameter optimization can be formulated as a black-box optimization problem. Because the inner-loop tuning objective has been specified in Equation (20) (i.e., using the $K_{\mathrm{in}}$-fold average ${\mathrm{WMSE}}_{t}$ as the criterion), the inner-loop cross-validation loss can be treated as a black-box objective function f(θ) of the hyperparameters θ, and the goal is to search for its minimum over Θ.

In terms of solution strategies, common approaches to hyperparameter search include grid search, random search, and sequential model-based optimization (SMBO). Because each function evaluation in this study requires model training and $K_{\mathrm{in}}$-fold validation, the cost is high; moreover, the objective function is typically non-differentiable and may be non-convex. Therefore, we adopt an SMBO framework and implement the acquisition-function mechanism commonly used in BO. BO approximates f(·) using a surrogate model and leverages an acquisition function to adaptively balance “exploration” of high-uncertainty regions and “exploitation” of low-mean regions, thereby approaching better hyperparameter configurations more efficiently under a limited evaluation budget [32,38].

#### 3.3.2. Surrogate Models and Uncertainty Modeling

In BO, we do not prespecify a single surrogate model. Instead, we independently run the hyperparameter search using three surrogate models: Gaussian process regression (GPR), a RF regressor (RF surrogate), and an extra-trees regressor (ET surrogate) [39]. Specifically, for a given outer fold *k* and a candidate learning algorithm A, BO is executed with each surrogate model to obtain three candidate optima,

(26) $$\theta_{\mathrm{GPR}}^{*(k)},\;\theta_{\mathrm{RF}}^{*(k)},\;\theta_{\mathrm{ET}}^{*(k)},$$

along with their corresponding best inner-loop validation losses,

(27) $$f_{\mathrm{GPR}}^{*(k)},\;f_{\mathrm{RF}}^{*(k)},\;f_{\mathrm{ET}}^{*(k)}.$$

We then select the configuration with the smallest validation loss among the three as the inner-loop optimal hyperparameters for the *k*-th outer fold:

(28) $$\theta^{*(k)}=\arg\min_{\theta \in \{\theta_{\mathrm{GPR}}^{*(k)},\theta_{\mathrm{RF}}^{*(k)},\theta_{\mathrm{ET}}^{*(k)}\}}f\left(\theta \right).$$

The motivation is that GPR is often sample-efficient for low-dimensional and relatively smooth objectives, whereas tree-ensemble surrogates are typically more robust for non-smooth objectives, mixed-type hyperparameters, or higher-dimensional spaces. The “multi-surrogate in parallel and select the best” strategy reduces the risk of suboptimal tuning due to mismatch of any single surrogate model.

For any surrogate model, at iteration *t* we fit the surrogate using the historical observations

(29) $$H_{t}={\left\{\left(\theta_{i},f\left(\theta_{i}\right)\right)\right\}}_{i=1}^{t},$$

and output the predictive mean and an uncertainty measure for a candidate point, denoted by $\left(\mu_{t}\left(\theta \right),\sigma_{t}\left(\theta \right)\right)$. For GPR, the posterior predictive distribution satisfies

(30) $$f\left(\theta \right)|H_{t}∼N\left(\mu_{t}\left(\theta \right),\sigma_{t}^{2}\left(\theta \right)\right),$$

where $\sigma_{t}^{2}\left(\theta \right)$ is the posterior variance. For RF/ET surrogates, $\mu_{t}\left(\cdot \right)$ and $\sigma_{t}\left(\cdot \right)$ can be obtained from the empirical mean and variance (or an approximate estimate) of the ensemble predictions. To be consistent with the computational forms of acquisition functions such as EI and PI, we adopt a Gaussian approximation in the acquisition computation:

(31) $$f\left(\theta \right)|H_{t}\approx N\left(\mu_{t}\left(\theta \right),\sigma_{t}^{2}\left(\theta \right)\right),$$

where $\sigma_{t}^{2}\left(\theta \right)$ denotes the uncertainty measure derived from the variance of the tree-ensemble distribution (or its approximation).

#### 3.3.3. Acquisition Function and Search Strategy

The acquisition function converts the question of “which hyperparameter configuration to evaluate next” into an explicitly optimizable problem. In our implementation, we consistently adopt gp_hedge provided by scikit-optimize as the acquisition strategy [39]. gp_hedge can be understood as a “hedging” strategy: at each iteration it considers multiple classical acquisition functions—expected improvement (EI), probability of improvement (PI), and lower confidence bound (LCB)—and adaptively selects the next evaluation point based on their historical performance, thereby achieving a robust trade-off between exploration and exploitation [39,40].

Formally, let the historical observation set at iteration *t* be given in Equation (29). The surrogate model provides a predictive mean and an uncertainty measure $\left(\mu_{t}\left(\theta \right),\sigma_{t}\left(\theta \right)\right)$ at a candidate point θ. In gp_hedge, a set of candidate acquisition functions {EI,PI,LCB} is maintained. At each iteration, gp_hedge constructs

(32) $$a_{t}^{\left(j\right)}\left(\theta \right),\;j\in \left\{\mathrm{EI},\mathrm{PI},\mathrm{LCB}\right\},$$

and obtains the candidate point suggested by each acquisition function:

(33) $$\theta_{t+1}^{\left(j\right)}=\arg\max_{\theta \in \Theta }a_{t}^{\left(j\right)}\left(\theta \right).$$

Then, gp_hedge weights the acquisition functions according to their historical “gains” (which can be interpreted as their contributions to improving the current best value) and probabilistically selects the final next evaluation point $\theta_{t+1}$ from $\left\{\theta_{t+1}^{\left(j\right)}\right\}$. Intuitively, this mechanism can automatically switch across stages: it tends to favor LCB when stronger exploration is needed and EI/PI when approaching the optimum region, thereby reducing sensitivity to manually choosing a single acquisition function.

In summary, at iteration *t*, gp_hedge adaptively determines the next hyperparameter point $\theta_{t+1}$ by hedging among {EI,PI,LCB}. We then evaluate $f(\theta_{t+1})$ via inner-loop cross-validation and update

(34) $$H_{t+1}=H_{t}\cup \left\{\left(\theta_{t+1},f\left(\theta_{t+1}\right)\right)\right\},$$

repeating the above procedure until the evaluation budget is reached or a convergence criterion is satisfied.

#### 3.3.4. Integration with Inner Cross-Validation

Within the NCV framework, BO is performed in the “inner loop”: for a fixed outer-fold training set $D_{\mathrm{train}}^{\left(k\right)}$, we conduct a $K_{\mathrm{in}}$-fold split within it. Each BO function evaluation corresponds to the $K_{\mathrm{in}}$-fold average validation loss in the inner loop (i.e., ${\mathrm{WMSE}}_{t}$ in the *t* domain). BO is run independently for each candidate surrogate model (GPR/RF/ET) to produce candidate optimal hyperparameters, and the final $\theta^{*(k)}$ is selected by choosing the configuration with the smallest inner-loop best validation loss.

### 3.4. Summary of Section 3

Based on the feature representation obtained after multidimensional equal-frequency binning, this section develops a unified “NCV+BO” modeling and tuning framework tailored to disturbance factors and a skewed target distribution. First, to strengthen learning on the low-yield risk region, we transform the target from yield *y* to the log-defect-rate domain $t=\log(1-y+\epsilon_{d})$ during training and inner-loop tuning, and introduce a fixed group-ratio weighting strategy based on the business threshold τ=0.995 so that low-yield samples receive stronger error penalties in the weighted loss. Second, we use an outer cross-validation with $K_{\mathrm{out}}=5$ folds to evaluate and select the best-performing ML algorithm, and within each outer fold we perform hyperparameter selection via inner cross-validation with $K_{\mathrm{in}}=5$ folds combined with BO. The final evaluation in the outer loop is reported in the yield domain *y*, using metrics including MSE, WMSE, MSE(y<τ), $R^{2}$, and Acc(α). In implementation, BO is executed independently with three surrogate models (GPR, RF, and ET), and within each outer fold the final hyperparameters are determined by selecting the surrogate-specific candidate that achieves the best inner-loop validation loss. The acquisition strategy uses gp_hedge to adaptively hedge among EI, PI, and LCB, improving tuning efficiency and stability under a limited evaluation budget. Overall, this section provides a complete and reproducible pipeline from target transformation and weighted learning to the “outer evaluation/inner selection” procedure, establishing a unified evaluation benchmark for the subsequent experimental results and model comparisons.

## 4. Results and Analysis

Based on the data preprocessing and feature-screening procedures in Section 2 and the NCV with BO (NCV+BO) framework in Section 3, this section evaluates the prediction consistency of four regression models (FNN, SVR, RF, and XGB) under the task definition of typical yield. We further report the final model selection, cross-validation results on the full dataset, and an interpretability analysis. To characterize both global fitting performance and predictive capability in the low-yield tail, we use the following metrics:

- MSE (Equation (12)): mean squared error on the full test set;
- WMSE (Equation (13)): weighted mean squared error computed with fixed weights, where the total weight assigned to the low-yield tail samples (y<99.5%) is 50%;
- MSE(y<99.5%) (Equation (14)): mean squared error on low-yield tail samples (y<99.5%), used to directly quantify tail prediction accuracy;
- $R^{2}$ (Equation (15)): coefficient of determination;
- Acc(0.5%) and Acc(0.2%) (Equation (16)): accuracy defined by the relative deviation $\left|\frac{y-\hat{y}}{y}\right|$, where a prediction is counted as accurate when it is below the threshold α (0.5% and 0.2%, respectively).

### 4.1. Comparison of Outer-Fold Results in NCV

Table 3 summarizes the test results of the five outer folds in NCV. Overall, all four models achieve approximately 0.92–0.94 in Acc(0.5%), whereas the differences become more pronounced under the stricter Acc(0.2%) criterion and the tail error metrics (WMSE and MSE(y<99.5%)). In terms of Mean ± Std across metrics, RF shows the strongest overall conditional performance: its average MSE is $\left(3.2\pm 2.9\right)\times 10^{-5}$, average WMSE is $\left(7.5\pm 6.9\right)\times 10^{-5}$, and average MSE(y<99.5%) is $\left(1.5\pm 1.4\right)\times 10^{-4}$, while achieving the highest average $R^{2}$ (0.65±0.092). These results indicate that RF attains a better balance between overall accuracy and low-yield tail accuracy.

The other models exhibit notable degradation on certain folds. For example, FNN attains an $R^{2}$ of only 0.079 on Fold_1, and SVR shows $R^{2}<0$ on Fold_1, suggesting that, in industrial scenarios with limited features and potential operating-condition drift, these models are more sensitive to data partitioning. XGB achieves a slightly higher average Acc(0.2%) (0.75±0.037), but its average $R^{2}$ is clearly lower than that of RF (0.52±0.22), and it performs relatively weakly on some folds (e.g., Fold_0); therefore, its overall advantage is inferior to RF.

**Figure 7:**
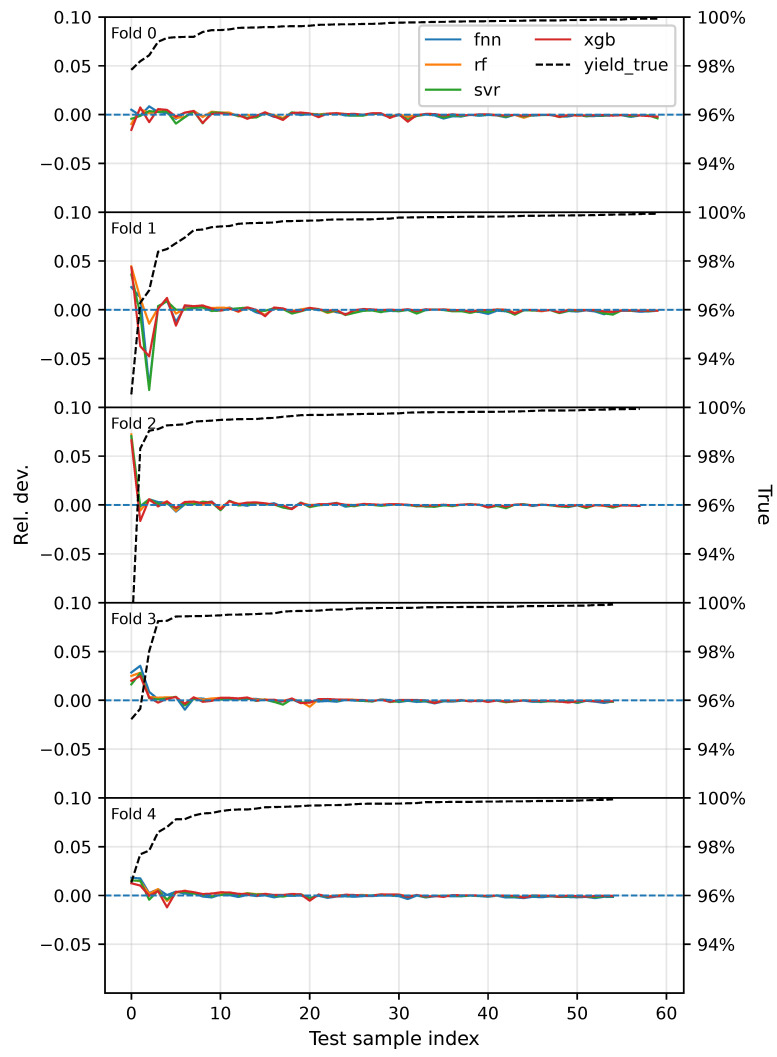
Results on the test set of each outer fold in cross-validation. The figure shows the distribution of relative deviation (Rel. dev.) of yield predictions for different models, as well as the distribution of ground-truth values (True) in the test set. Samples are ordered by ascending ground-truth yield.

To more intuitively examine how errors distribute across different yield ranges, Figure 7 shows the distribution of the relative deviation $\left|\frac{y-\hat{y}}{y}\right|$ for each model on the outer-fold test sets. It can be observed that, even with the fixed sample-weighting strategy, prediction accuracy for low-yield samples remains markedly lower than that in the high-yield region, with Fold_1 and Fold_2 being the most prominent. This suggests that the low-yield region is often accompanied by stronger operating-condition drift or unobserved disturbances. Although weighting increases the model’s attention to low-yield samples, it cannot fully compensate for the scarcity of information and the increased distributional complexity in the low-yield region.

The figure also indicates that RF achieves smaller relative deviations and better stability in the low-yield region, which is consistent with its lower WMSE and MSE(y<99.5%) in Table 3. This finding suggests that RF provides stronger robustness and better applicability for the industrial data scenario considered in this study.

### 4.2. Final Model Selection and Five-Fold Cross-Validation on the Full Dataset

Considering global accuracy (MSE), tail accuracy (MSE(y<99.5%)), weighted error (WMSE), goodness of fit ($R^{2}$), and the stricter accuracy metric Acc(0.2%) across outer folds, we select RF as the final model. To characterize fold-to-fold stability under the final training protocol and to determine the final hyperparameter configuration, we perform five-fold cross-validation on the full dataset. The results are reported in Table 4. Overall, the model shows stable performance on the global and strict accuracy metrics, whereas the tail errors still exhibit some fold-to-fold variation, consistent with the low-yield prediction challenges revealed in Figure 7.

Table 5 reports the optimal RF hyperparameter configuration obtained under five-fold cross-validation. This configuration is identified by BO through iterative search within the predefined hyperparameter space. For each candidate hyperparameter setting, five-fold cross-validation is used to compute the average WMSE, and the final configuration is selected as the one that achieves the best performance under the five-fold cross-validation.

### 4.3. SHAP-Based Interpretability Analysis

To quantify the contribution of different process features to yield prediction and to enhance interpretability and usability, we trained the final RF model on the complete set of 288 operating condition dictionary samples using the hyperparameters in Table 5, and performed SHAP (SHapley Additive exPlanations) analysis [41]. SHAP is based on Shapley values from cooperative game theory and decomposes the prediction of an individual sample into a “baseline value and sum of feature contributions”, providing consistent and additive explanations for the impact of each feature on each sample.

**Figure 8:**
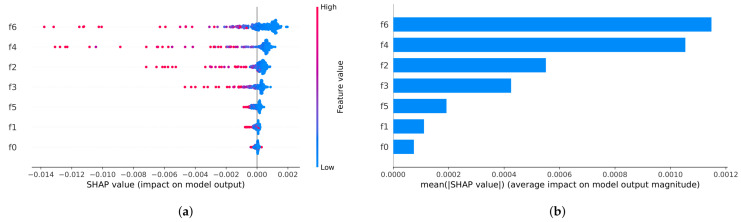
SHAP interpretability results for the RF model. (**a**) SHAP summary beeswarm plot: sample-level distribution of feature impacts on predictions; (**b**) SHAP bar plot: global feature importance ranked by mean absolute SHAP values.

Figure 8 presents two types of global explanations:

- SHAP summary beeswarm plot: The horizontal axis shows SHAP values, indicating the direction and magnitude by which a feature drives the model output. Each point corresponds to the contribution of that feature for one sample, and the color encodes the feature value from low to high. A wider horizontal spread of points indicates a stronger influence on the model output and greater heterogeneity across samples.
- SHAP feature importance bar plot: Features are ranked by the mean absolute SHAP value (mean |SHAP|), reflecting their global importance and helping identify key process variables that should be prioritized for monitoring.

By jointly examining these two plots, we can further analyze the directional effects of key features (i.e., how high/low feature values correspond to positive/negative SHAP contributions) as well as potential nonlinearities and interactions, thereby providing evidence for mechanism-oriented analysis of low-yield risk samples and for process parameter adjustment.

### 4.4. Summary of Section 4 and Practical Implications

In summary, under the fixed sample-weighting setting (where the low-yield tail accounts for 50% of the total weight), RF shows the strongest overall conditional performance and relatively good stability in both the outer-loop NCV and the five-fold cross-validation on the full dataset, particularly on the tail-focused metrics (WMSE and MSE(y<99.5%)). However, the relative-deviation distributions still show that prediction is substantially more challenging in the low-yield region (most prominently in Fold_1 and Fold_2), suggesting that low-yield samples may involve stronger operating-condition drift, unobserved disturbances, or more complex nonlinear mechanisms. Future improvements may focus on two directions: enhancing the representational capacity for low-yield operating conditions (e.g., incorporating additional sensor signals or constructing more discriminative stability/drift features) and risk-oriented output strategies (e.g., uncertainty quantification, interval prediction, or alarm-threshold optimization), to further improve reliability in engineering deployment.

## 5. Discussion

### 5.1. Ablation Study Design and Comparison Dimensions

To validate the necessity of key design choices in our framework and to clarify their applicability boundaries, we conduct ablation studies centered on the multidimensional equal-frequency binning strategy and the low-yield sample-weighting mechanism. Specifically, the ablations compare two dimensions:

(1) Binning strategy (granularity of feature-space partitioning): We consider three ways of organizing the data:

- 288 bins: Multidimensional equal-frequency binning of the feature space, where f2 and f6 use 3 bins and the remaining features use 2 bins;
- 432 bins: A finer-grained binning setting, where f2, f4, and f6 use 3 bins and the remaining features use 2 bins;
- all samples (no binning): No binning is performed; all 5209 batch samples are used to directly predict the per-batch observed yield.

Here, f6, f2, and f4 are the top three features ranked by mutual information (from high to low; see Table 1), whereas f6, f4, and f2 are the top three features ranked by mean SHAP values (from high to low; see Figure 8). Therefore, the 288-bin and 432-bin settings form a targeted comparison in terms of whether more important features are included as finer-grained partitioning axes.

(2) Sample weighting (risk-oriented cost-sensitive learning): Under each binning configuration, we further compare “with sample weights (W/weights)” versus “without sample weights (W/O weights)”. When weighting is enabled, the training objective is explicitly guided to improve fitting on low-yield samples, aligning with quality-risk management scenarios. The unweighted setting serves as a baseline to assess the gains and trade-offs introduced by the weighting mechanism.

Table 6 summarizes the outer-fold (NCV) results of four modeling methods (FNN, RF, SVR, and XGB) in terms of mean ± std. The evaluation metrics include overall error (MSE, WMSE), low-yield subset error (MSE(y<99.5%)), goodness of fit ($R^{2}$), and threshold accuracy (Acc(0.5%), Acc(0.2%)). Based on these results, we discuss the performance changes associated with the binning and weighting designs, and summarize the advantages and limitations of the proposed framework.

### 5.2. Interpreting the Ablation Results: When Binning and Weighting Help (And When They Do Not)

Table 6 compares two key design dimensions: the binning strategy (288/432/no binning) and sample weighting (with/without). Overall, three empirical patterns can be summarized.

(1) Without binning (all samples), the error scale increases sharply, reflecting the statistical mismatch between “per-batch observed yield” and “typical yield”. Under the “no binning and weighting” setting, MSE/WMSE/MSE(y<99.5%) of all four models increase to the $10^{-3}$ order of magnitude, whereas the binning-based setting (with the operating condition dictionary and the typical yield target definition) keeps these metrics around the $10^{-5}$ level, i.e., approximately two orders of magnitude lower. This gap stems from a redefinition of the learning target and the resulting change in label variance. Without binning, the model directly performs point prediction for “per-batch observed yield”, whose labels contain substantial unexplained fluctuations (e.g., unobserved disturbances and latent-state drift). With a limited set of observable features, it is difficult to stably approximate such a high-variance target; sample weighting changes the loss emphasis but does not reduce the label variance induced by unobserved disturbances. In contrast, the binning strategy partitions the feature space and uses a robust within-bin representative (e.g., the median) to characterize the operating-condition central tendency. This effectively aggregates observed yields under the same observable operating condition, transforming the learning problem into predicting a smoother and more identifiable “typical yield”.

Moreover, in real production line data, yield is highly skewed (high-yield samples dominate), and feature-space coverage is uneven, exhibiting dense and sparse regions. Under the per-batch point-prediction definition, this uneven coverage makes the optimization more likely to be dominated by samples from dense regions. Combined with high label variance, this leads to larger fold-to-fold fluctuations. Therefore, sample weighting alone cannot simultaneously address both difficulties: a high-variance target and uneven feature-space coverage. By contrast, multidimensional equal-frequency binning provides an operating condition stratification and statistical aggregation mechanism aligned with the typical yield definition, yielding a more stable data organization and more robust conditional evaluation.

(2) 288 bins outperform 432 bins, indicating an optimal range of binning granularity; overly fine binning increases variance and instability. When switching from 288 to 432 bins, FNN and SVR show markedly negative $R^{2}$ values with very large variance (substantial fluctuations across outer folds), and RF and XGB also exhibit a sharp increase in the variance of $R^{2}$. This behavior is consistent with the bias–variance trade-off: finer binning reduces the number of samples per cell, which can lead to (i) insufficient statistical representativeness within each partition, (ii) greater distribution shift at the partition level across train/validation splits, and (iii) further amplification of low-yield samples within a small number of cells after weighting, potentially causing numerical instability or overfitting. These results suggest that finer binning is not always better; instead, the binning granularity should satisfy a dual requirement: sufficient sample support within each cell and adequate coverage of the key heterogeneity in the data. Under the current data scale, 288 bins appear closer to this balance point.

(3) Weighting generally improves tail-region errors, but it introduces a visible cost in the high-yield region. Comparing “288 bins and weighting” with “288 bins without weighting”, multiple models achieve better MSE(y<99.5%) (tail-subset error), indicating that weighting indeed shifts the optimization focus from the dominant high-yield region to the higher-risk low-yield region. However, stricter threshold metrics such as Acc(0.2%) slightly decrease for some models, reflecting a typical trade-off: when model capacity and feature information are limited, improving robustness in the low-yield region often comes at the expense of peak precision in the high-yield region. From an industrial perspective, this trade-off is often acceptable because low-yield batches correspond to higher quality risk and larger economic losses; however, it also implies that the weighting strength and the metric set should be selected according to specific business objectives.

### 5.3. Advantages of the Proposed Framework: Robustness and Interpretability for Industrial Deployment

Synthesizing the ablation results, the main advantages of the proposed framework can be summarized as follows.

(1) The combination of binning and weighting aligns the learning objective with risk-oriented requirements. Industrial yield distributions are highly skewed: high-yield samples dominate, whereas the low-yield tail is scarce but associated with higher risk. If one directly minimizes MSE on the raw samples, optimization tends to be dominated by the high-yield region, resulting in insufficient fitting in the low-yield region. In our framework, multidimensional equal-frequency binning first organizes samples into locally homogeneous operating condition units, allowing training to focus on the observable operating-condition structure and reducing the influence of extreme fluctuations on global learning. Sample weighting then explicitly biases the loss contribution toward the low-yield region, making the optimization objective consistent with quality-risk management goals. Compared with using only weighting or only resampling, the combination of “binning (stable operating-condition representation) and weighting (explicit risk preference)” generally improves tail-region errors and yields more consistent tail-oriented fitting across folds, although residual fold-to-fold variation remains in the low-yield region.

(2) Consistent gains across models indicate generality and transferability. The ablation study shows that FNN, RF, SVR, and XGB are generally better or more stable under the 288-bin setting. This suggests that the improvement does not rely on a specific model structure, but rather reflects a data-centric strategy for industrial data: reorganizing the data and redefining the learning target. Binning provides a unified operating-condition stratified representation, and weighting injects business risk preference in a consistent manner. As a result, the framework can be flexibly reused across different algorithmic choices and computational constraints. For deployment, such model-agnostic behavior reduces the cost of migration and iteration and makes the approach easier to extend to different production lines and modeling preferences.

(3) An interpretability loop facilitates integration with process knowledge and actionable outputs. Industrial deployment requires not only accuracy but also interpretability, traceability, and operational actionability. We use statistical dependence measures such as mutual information to support global screening and the selection of partitioning axes, ensuring that variables entering the binning and modeling stages exhibit stable associations. We then use SHAP to provide model-level local contribution explanations, revealing which features drive predictions toward lower yield within specific operating condition units, and whether dominant factors shift across operating conditions. This yields a closed-loop chain of “statistical dependence–model contribution–process validation”: on the one hand, high-risk operating conditions can be mapped back to concrete parameter ranges and equipment-status combinations to narrow down troubleshooting; on the other hand, the results provide data-driven evidence for narrowing process windows and for post hoc engineering review, thereby improving the credibility and usability of the model in continuous production line operation.

### 5.4. Positioning Relative to Prior Studies

Compared with many previous VM and related quality-prediction studies, the present study addresses a setting characterized by partial observability. Key influencing factors are incompletely measured or cannot be consistently aligned to individual samples, and the observed batch yield therefore contains substantial fluctuations induced by unobserved disturbances. This condition differs from that in many prior studies conducted under relatively more complete observability (as discussed in Section 1), making direct quantitative comparison with published results not strictly appropriate.

Accordingly, the contribution of the present study lies not in a universal methodological improvement, but in a problem-specific formulation based on operating-condition-level typical yield prediction and multidimensional equal-frequency stratification, which provides a more stable basis for in-line yield modeling than direct prediction on unbinned batch samples.

### 5.5. Limitations and Future Directions

The ablation study also reveals boundary conditions and potential risks of the framework.

(1) Binning granularity is sensitive to sample size; overly fine binning can induce high variance and negative $R^{2}$: The pronounced instability under 432 bins indicates that when the number of samples per cell is insufficient, or when low-yield samples are extremely unevenly distributed across cells, binning may shift from a distribution-alignment mechanism to an uncertainty-amplifying procedure. In addition, if key features exhibit strong interactions or collinearity, the model becomes more sensitive to the coverage of the joint distribution. Finer binning reduces the effective sample size in each joint subspace (and weighting further amplifies the influence of a few samples), thereby increasing estimation variance and causing cross-fold instability. Future work may incorporate minimum-sample constraints per cell and density-aware adaptive merging to improve stability.

(2) The fixed weighting scheme should be recalibrated to specific business objectives: The current weighting emphasizes low-yield risk, but it may sacrifice high-yield precision under certain models or thresholds (e.g., Acc(0.2%)). With more data, it would be valuable to explore piecewise weighting schemes (stratified by yield intervals) to achieve a more controllable multi-objective trade-off.

(3) The typical yield definition implies a conditional evaluation protocol: We define typical yield using within-bin medians after binning and construct the operating condition dictionary accordingly. This design extracts a more predictable central tendency under incomplete features, but it also implies that evaluation is conditional on a fixed dictionary: The bin boundaries and within-bin medians are estimated once using the full dataset, and the outer folds do not explicitly account for the uncertainty introduced by dictionary reconstruction. With larger sample sizes, a more deployment-oriented evaluation could be conducted in two ways. One is a time-extrapolation protocol, in which the dictionary is constructed using a historical window and validated on a subsequent future window. The other is to reconstruct the binning and representative dictionary entries within each nested-CV fold, so as to obtain more conservative performance estimates.

## 6. Conclusions and Outlook

This study addresses the yield-prediction needs in electronic component manufacturing by developing a complete VM-inspired pipeline covering data cleaning, feature screening, model training, evaluation, and interpretation. Under process-mechanism constraints, we remove variables that are not physically related to the visual inspection outcome and obtain a structured feature set suitable for modeling. To mitigate unobserved disturbances caused by incomplete feature coverage in industrial data, we introduce a multidimensional equal-frequency binning strategy that constructs approximately balanced partitions under multiple conditioning features and focuses learning on the operating-condition central tendency. This design reduces the sensitivity of prediction to unobserved perturbations in a statistical sense and provides a consistent stratified basis for subsequent robust evaluation. Given the highly concentrated yield distribution, where low-yield samples are rare yet associated with higher risk, we adopt a fixed weighting strategy so that low-yield samples receive a larger overall weight during training, and we establish a multi-metric evaluation protocol that includes both global errors and low-yield subset errors. Within the NCV+BO framework, we systematically compare FNN, SVR, XGB, and RF, obtaining conditional performance estimates and cross-fold consistency conclusions under a fixed operating condition dictionary. Furthermore, using SHAP-based interpretability analysis, we quantify the contributions of key features to predictions from both global and local perspectives, providing evidence to support quality-risk warning and root-cause analysis and enhancing the communicability and usability of the model for production line deployment.

Due to limitations in (i) the overall sample size and coverage of the joint feature space, (ii) the scarcity of low-yield tail samples, and (iii) the time span of online sequential data and the conditions required for closed-loop deployment, this work has not fully explored finer-grained interaction modeling, adaptive binning, recalibration of weighting strategies, or more rigorous deployment-oriented evaluation. In addition, potential interaction effects and collinearity among key features may further reduce the effective sample size in joint subspaces under small-sample conditions, amplifying local sparsity and estimation uncertainty and weakening stability guarantees for fine-grained partitions. As more data are continuously collected to expand sample size and cover more operating conditions, future work can extend this framework in several directions. First, regarding the stability boundary of binning granularity, adaptive granularity [42] and stability constraints (e.g., minimum samples per cell, density-aware adaptive merging, and smoothing regularization) [43] can be incorporated into multidimensional equal-frequency binning. Combined with tools such as SHAP interaction effects, this may enable more reliable characterization of feature interactions and provide principled guidance for selecting binning granularity and feature combinations. Second, once more tail samples become available, piecewise weighting strategies stratified by yield intervals and multi-objective weight calibration can be explored to achieve a controllable trade-off between low-yield risk identification and high-yield precision. Third, to reduce the conditional nature induced by the typical yield task definition, a time-extrapolation evaluation protocol can be adopted, in which the operating condition dictionary is constructed on a historical window and validated on a subsequent future window; alternatively, the binning and representative samples can be reconstructed within each nested-CV fold to more explicitly account for dictionary-reconstruction uncertainty, yielding more conservative and deployment-aligned performance estimates. Finally, once online data streams and operational conditions are available, the framework can be extended with drift monitoring and incremental updating [44], and uncertainty quantification (e.g., conformal prediction [45]) can be integrated to output calibrated risk intervals, improving the operability of alarm-threshold setting and engineering response.

## Figures and Tables

**Table 1:** Statistical correlation analysis results between features and yield.

Feature ID	Pearson	Spearman	Mutual Information
f0	−0.098388	−0.161045	0.007292
f1	−0.085778	−0.176422	0.026217
f2	−0.199646	−0.271310	0.046599
f3	−0.242725	−0.096987	0.024621
f4	−0.184464	−0.288616	0.046569
f5	−0.078494	−0.247221	0.042845
f6	−0.169933	−0.340402	0.065161

**Table 2:** Number of bins for each feature in multidimensional equal-frequency binning.

	f0	f1	f2	f3	f4	f5	f6	Total
Bins	2	2	3	2	2	2	3	288

**Table 3:** Outer-fold results of NCV. Reported metrics include MSE, WMSE, MSE(y<99.5%), $R^{2}$, Acc(0.5%), and Acc(0.2%).

Model	Fold	MSE	WMSE	MSE(y<99.5%)	$R^{2}$	Acc(0.5%)	Acc(0.2%)
FNN	0	$4.8\times 10^{-6}$	$7.9\times 10^{-6}$	$1.3\times 10^{-5}$	0.73	0.97	0.70
	1	$1.1\times 10^{-4}$	$2.8\times 10^{-4}$	$5.5\times 10^{-4}$	0.079	0.90	0.60
	2	$7.2\times 10^{-5}$	$1.7\times 10^{-4}$	$3.4\times 10^{-4}$	0.53	0.95	0.71
	3	$3.9\times 10^{-5}$	$9.5\times 10^{-5}$	$1.9\times 10^{-4}$	0.45	0.93	0.82
	4	$1.4\times 10^{-5}$	$3.2\times 10^{-5}$	$6.2\times 10^{-5}$	0.62	0.95	0.73
	Mean ± Std	$\left(4.9\pm 4.5\right)\times 10^{-5}$	$\left(1.2\pm 1.1\right)\times 10^{-4}$	$\left(2.3\pm 2.2\right)\times 10^{-4}$	0.48±0.25	0.94±0.025	0.71±0.078
RF	0	$4.8\times 10^{-6}$	$9.4\times 10^{-6}$	$1.7\times 10^{-5}$	0.73	0.97	0.70
	1	$3.9\times 10^{-5}$	$9.2\times 10^{-5}$	$1.8\times 10^{-4}$	0.69	0.90	0.70
	2	$7.8\times 10^{-5}$	$1.9\times 10^{-4}$	$3.7\times 10^{-4}$	0.50	0.93	0.74
	3	$2.6\times 10^{-5}$	$6.3\times 10^{-5}$	$1.2\times 10^{-4}$	0.63	0.95	0.76
	4	$1.1\times 10^{-5}$	$2.6\times 10^{-5}$	$5.0\times 10^{-5}$	0.70	0.91	0.80
	Mean ± Std	$\left(3.2\pm 2.9\right)\times 10^{-5}$	$\left(7.5\pm 6.9\right)\times 10^{-5}$	$\left(1.5\pm 1.4\right)\times 10^{-4}$	0.65±0.092	0.93±0.027	0.74±0.043
SVR	0	$6.4\times 10^{-6}$	$1.2\times 10^{-5}$	$2.0\times 10^{-5}$	0.63	0.95	0.65
	1	$1.3\times 10^{-4}$	$3.2\times 10^{-4}$	$6.3\times 10^{-4}$	−0.049	0.92	0.67
	2	$7.4\times 10^{-5}$	$1.8\times 10^{-4}$	$3.5\times 10^{-4}$	0.52	0.95	0.71
	3	$2.0\times 10^{-5}$	$4.7\times 10^{-5}$	$9.2\times 10^{-5}$	0.72	0.95	0.78
	4	$1.1\times 10^{-5}$	$2.4\times 10^{-5}$	$4.8\times 10^{-5}$	0.71	0.95	0.84
	Mean ± Std	$\left(4.8\pm 5.3\right)\times 10^{-5}$	$\left(1.2\pm 1.3\right)\times 10^{-4}$	$\left(2.3\pm 2.6\right)\times 10^{-4}$	0.51±0.32	0.94±0.014	0.73±0.079
XGB	0	$1.1\times 10^{-5}$	$2.3\times 10^{-5}$	$4.2\times 10^{-5}$	0.37	0.88	0.70
	1	$9.4\times 10^{-5}$	$2.3\times 10^{-4}$	$4.6\times 10^{-4}$	0.24	0.90	0.73
	2	$7.0\times 10^{-5}$	$1.7\times 10^{-4}$	$3.3\times 10^{-4}$	0.55	0.95	0.76
	3	$1.9\times 10^{-5}$	$4.6\times 10^{-5}$	$9.0\times 10^{-5}$	0.73	0.96	0.75
	4	$9.9\times 10^{-6}$	$2.2\times 10^{-5}$	$4.3\times 10^{-5}$	0.73	0.93	0.80
	Mean ± Std	$\left(4.1\pm 3.9\right)\times 10^{-5}$	$\left(9.7\pm 9.5\right)\times 10^{-5}$	$\left(1.9\pm 1.9\right)\times 10^{-4}$	0.52±0.22	0.92±0.033	0.75±0.037

**Table 4:** Final 5-fold cross-validation results of RF on the complete operating condition dictionary dataset.

Fold	MSE	WMSE	MSE(y<99.5%)	$R^{2}$	Acc(0.5%)	Acc(0.2%)
0	$3.7\times 10^{-6}$	$6.9\times 10^{-6}$	$1.2\times 10^{-5}$	0.77	0.98	0.82
1	$4.6\times 10^{-5}$	$1.1\times 10^{-4}$	$2.2\times 10^{-4}$	0.62	0.92	0.70
2	$8.9\times 10^{-5}$	$2.1\times 10^{-4}$	$4.2\times 10^{-4}$	0.42	0.95	0.69
3	$3.0\times 10^{-5}$	$7.4\times 10^{-5}$	$1.5\times 10^{-4}$	0.57	0.96	0.76
4	$1.4\times 10^{-5}$	$3.3\times 10^{-5}$	$6.5\times 10^{-5}$	0.62	0.95	0.80
Mean ± Std	(3.7±3.3) $\times 10^{-5}$	(8.7±8.0) $\times 10^{-5}$	(1.7±1.6) $\times 10^{-4}$	0.60± 0.12	0.95± 0.025	0.75± 0.057

**Table 5:** Optimal RF hyperparameter configuration selected on the complete operating condition dictionary dataset.

Item	Value
max_depth	36
max_features	7
min_samples_leaf	3
min_samples_split	4
n_estimators	1439
random_state	51

**Table 6:** Outer-fold comparison of four modeling methods (FNN, RF, SVR, and XGB) under four settings. The four settings are “W/weights 288 bins”, “W/weights 432 bins”, “W/weights all samples”, and “W/O weights 288 bins”. In the all-samples (no-binning) setting, the target is the per-batch observed yield, whereas the binning-based settings use the typical yield task definition.

Model	Set	MSE	WMSE	MSE(y<99.5%)	$R^{2}$	Acc(0.5%)	Acc(0.2%)
FNN	**W/weights288 bins**	** (4.9±4.5) $\times 10^{-5}$ **	** (1.2±1.1) $\times 10^{-4}$ **	** (2.3±2.2) $\times 10^{-4}$ **	** 0.48± ** ** 0.25 **	** 0.94± ** ** 0.025 **	** 0.71± ** ** 0.078 **
	W/weights432 bins	(1.2±1.2) $\times 10^{-4}$	(2.8±2.9) $\times 10^{-4}$	(5.5±5.8) $\times 10^{-4}$	−1.7± 5.0	0.92± 0.030	0.66± 0.036
	W/weightsall samples	(1.6±0.57) $\times 10^{-3}$	(2.2±0.79) $\times 10^{-3}$	(4.3±1.6) $\times 10^{-3}$	0.075± 0.028	0.71± 0.011	0.41± 0.017
	W/O weights288 bins	(5.6±4.2) $\times 10^{-5}$	(5.6±4.2) $\times 10^{-5}$	(2.7±2.1) $\times 10^{-4}$	0.37± 0.14	0.93± 0.029	0.75± 0.063
RF	**W/weights288 bins**	** (3.2±2.9) $\times 10^{-5}$ **	** (7.5±6.9) $\times 10^{-5}$ **	** (1.5±1.4) $\times 10^{-4}$ **	** 0.65± ** ** 0.092 **	** 0.93± ** ** 0.027 **	** 0.74± ** ** 0.043 **
	W/weights432 bins	(6.0±4.5) $\times 10^{-5}$	(1.4±1.1) $\times 10^{-4}$	(2.8±2.2) $\times 10^{-4}$	0.25± 0.71	0.93± 0.025	0.76± 0.053
	W/weightsall samples	(1.5±0.56) $\times 10^{-3}$	(2.1±0.78) $\times 10^{-3}$	(4.1±1.6) $\times 10^{-3}$	0.13± 0.033	0.67± $7.2\times 10^{-3}$	0.40± 0.025
	W/O weights288 bins	(3.8±3.3) $\times 10^{-5}$	(3.8±3.3) $\times 10^{-5}$	(1.8±1.6) $\times 10^{-4}$	0.58± 0.14	0.94± 0.037	0.76± 0.053
SVR	**W/weights288 bins**	** (4.8±5.3) $\times 10^{-5}$ **	** (1.2±1.3) $\times 10^{-4}$ **	** (2.3±2.6) $\times 10^{-4}$ **	** 0.51± ** ** 0.32 **	** 0.94± ** ** 0.014 **	** 0.73± ** ** 0.079 **
	W/weights432 bins	(1.1±1.5) $\times 10^{-4}$	(2.7±3.6) $\times 10^{-4}$	(5.3±7.1) $\times 10^{-4}$	−2.1± 6.1	0.92± 0.027	0.74± 0.033
	W/weightsall samples	(1.5±0.55) $\times 10^{-3}$	(2.0±0.76) $\times 10^{-3}$	(4.0±1.5) $\times 10^{-3}$	0.14± 0.048	0.69± $6.7\times 10^{-3}$	0.37± $7.0\times 10^{-3}$
	W/O weights288 bins	(1.1±0.80) $\times 10^{-4}$	(1.1±0.80) $\times 10^{-4}$	(5.9±4.2) $\times 10^{-4}$	−1.9± 3.7	0.92± $8.9\times 10^{-3}$	0.77± 0.046
XGB	**W/weights288 bins**	** (4.1±3.9) $\times 10^{-5}$ **	** (9.7±9.5) $\times 10^{-5}$ **	** (1.9±1.9) $\times 10^{-4}$ **	** 0.52± ** ** 0.22 **	** 0.92± ** ** 0.033 **	** 0.75± ** ** 0.037 **
	W/weights432 bins	(4.5±2.2) $\times 10^{-5}$	(1.0±0.56) $\times 10^{-4}$	(2.0±1.1) $\times 10^{-4}$	0.28± 0.91	0.90± 0.034	0.73± 0.050
	W/weightsall samples	(1.5±0.57) $\times 10^{-3}$	(2.0±0.79) $\times 10^{-3}$	(3.9±1.6) $\times 10^{-3}$	0.14± 0.044	0.62± 0.013	0.34± 0.014
	W/O weights288 bins	(3.5±2.9) $\times 10^{-5}$	(3.5±2.9) $\times 10^{-5}$	(1.6±1.4) $\times 10^{-4}$	0.57± 0.088	0.93± 0.013	0.74± 0.042

Note: The **bold** values in the “W/weights, 288 bins” rows correspond to the results reported in Table 3.

## Data Availability

The raw data supporting the conclusions of this article will be made available by the authors on request. The data are not publicly available due to commercial confidentiality obligations under the authors’ collaboration agreement.

## Abbreviations

The following abbreviations are used in this manuscript:

VM	virtual metrology
MLCCs	multilayer ceramic capacitors
ML	machine learning
NG	No Good
CCD	charge-coupled device
PU	positive unlabeled
NCV	nested cross-validation
BO	Bayesian optimization
FNN	feedforward neural network
XGB	XGBoost
RF	random forest
SVR	support vector regression
SMBO	sequential model-based optimization
GPR	Gaussian process regression
ET	extra-trees regressor
EI	expected improvement
PI	probability of improvement
LCB	lower confidence bound
MSE	mean squared error
WMSE	weighted mean squared error
SHAP	Shapley additive explanations
CMP	chemical mechanical planarization
PCA	principal component analysis
KSG	Kraskov–Stögbauer–Grassberger
MLP	multilayer perceptron
